# Role of rs2282679 in 25-hydroxyvitamin D levels and insulin resistance after a sleeve gastrectomy

**DOI:** 10.1038/s41387-024-00272-8

**Published:** 2024-04-03

**Authors:** Daniel de Luis, Olatz Izaola, David Primo, Juan José López Gómez, David Pacheco

**Affiliations:** 1 Endocrinology and Nutrition Research Center, School of Medicine, Valladolid, Spain; 2 Department of Endocrinology and Nutrition, Hospital Clinico Universitario, University of Valladolid, Valladolid, Spain

**Keywords:** Endocrinology, Genetics

## Abstract

**Background & aims:**

Some studies have reported links between 25-hydroxyvitamin D levels and the presence of obesity and some genetic variants. The aim of our design was to evaluate the effects of rs2282679 genetic variant of *CG* gene on 25-hydroxyvitamin D levels, weight loss and metabolic parameters after a robotic sleeve gastrectomy in premenopausal females with obesity.

**Methods:**

76 participants were enrolled. 25-hydroxyvitamin D levels, biochemical evaluation and anthropometric parameters were registered before surgery and after 3, 6 and 12 months follow up. Genotype of rs2282679 *CG* gene was evaluated.

**Results:**

The improvements in anthropometric parameters, blood pressure and lipid profile were similar in both genotypes (TT vs TG + GG). Basal insulin levels and HOMA-IR were greater in G allele carriers than non-carriers (Delta: 6.7 ± 1.2 mUI/L; *p* = 0.01) and (Delta: 1.3 ± 0.1 units; *p* = 0.02). 25-hydroxyvitamin D levels were lower in G allele carriers than non-carriers (Delta: 8.1 ± 1.1 ng/dl; *p* = 0.03). The levels of insulin and HOMA-IR remained greater in G allele carriers than non-carriers throughout all the visits. The levels of 25-hydroxyvitamin D remained lower in G allele carriers than non-G allele. The average level of 25-hydroxyvitamin D at 12 months in non-G allele carriers were above 30 ng/dl (36.0 ± 3.1 ng/dl) and the level in G allele carriers were below (24.9 ± 4.9 ng/dl).

**Conclusions:**

rs 2282679 (*GC*) was related with low 25 hydroxyvitamin D levels and insulin resistance. In addition, the presence of G allele produced a decrease in the improvement of 25-hydroxyvitamin D levels and insulin resistance after weight loss during 12 months.

## Introduction

Vitamin D has a lot of important metabolic functions beyond regulation of bone metabolism and calcium homeostasis. Moreover, Vitamin D deficiency is a prevalent clinical situation [1]. It is believed that this shortage may be connected to environmental elements (sun exposure, diet, and fatness) as well as the interplay between these factors and genetic components. It is well known, that low serum levels of 25-hydroxyvitamin D, a marker of circulating vitamin D levels, is associated with obesity, insulin resistance and diabetes mellitus [2].

Obesity is a complex condition influenced by various factors such as physical activity, dietary habits, and genetics [3, 4]. Moreover, genetic factors may affect up to 30-50% of the 25-hydroxyvitamin D levels depending on environmental factors [5, 6], too. Some single nucleotide variants (SNVs) located on *GC* gene that encode vitamin D-binding protein (DBP) have been related with 25-hydroxyvitamin D levels [6]. This transport protein is one of the many regulators of serum vitamin D levels, with approximately 80-90% of the total circulating 25-hydroxyvitamin D reservoir being bound to vitamin D-binding protein [7]. DBP is synthesized in the liver, and estrogens control its synthesis. One described *GC* gene variant is the rs2282679 polymorphism, located at 3´untranslated region (3´UTR) of the *GC* gene, which is involved in the modulation of this gene and related with 25-hydroxyvitamin D levels [8, 9].

Finally, significant evidence indicates that obesity is linked to a variety of metabolic abnormalities and conditions, including hypertension, type 2 diabetes mellitus, hyperlipidemia, cardiovascular diseases, cancer. Additionally, individuals with obesity often experience low levels of 25-hydroxyvitamin D. A weight loss of at least 5%, achieved through dietary changes or medical interventions such as bariatric surgery, can be beneficial for people with obesity; however, bariatric surgery [10] stands out as the primary approach known for producing substantial and lasting weight loss in individuals with severe obesity. One of these bariatric surgery techniques is sleeve gastrectomy [11]. Some differences in weight reduction and additional metabolic benefits following bariatric surgery might be associated with environmental elements (physical activity, dietary habits, frequency of visits to the Nutrition Unit, etc.) as well as the genetic predisposition of the patients [12]. Within this framework, women with obesity before menopause are at a high risk for lacking vitamin D [13]. There are no observational studies in the literature that have assessed the association between rs2282679 of the CG gene, 25-hydroxyvitamin D levels, and metabolic parameters linked to obesity in premenopausal women with obesity following bariatric surgery.

The aim of our design was to evaluate the effects of rs2282679 genetic variant of *CG* gene on 25-hydroxyvitamin D levels, weight loss and metabolic parameters after a robotic sleeve gastrectomy in premenopausal Caucasian females with obesity.

## Methods

### Population, inclusion and exclusion criteria

A sleeve gastrectomy surgery and follow-up appointments were conducted at the Interdisciplinary Obesity Unit of Valladolid University Hospitals. The study enrolled seventy-six premenopausal women with obesity who underwent sleeve gastrectomy under the supervision of the same medical team. Prior to enrollment, all participants were briefed about the protocol and provided written informed consent. The research protocol was approved by the Ethics Committee of HCUVa in Valladolid, Spain (Committee 19/1080) and adhered to the principles outlined in the Declaration of Helsinki.

The inclusion criteria were: females over 20 years of age, body mass index (BMI) > 40 kg/m^2^ or > 35 kg/m^2^ with associated comorbidities and presence of regular menstrual cycles with a follicle-stimulating hormone below 30 UI/L. The exclusion criteria were: active alcoholism, uncontrolled hypothyroidism, active Cushing syndrome, genetic syndromes associated with obesity, coagulopathy, neoplasia, renal or liver failure and use of corticosteroids.

### General procedures

The rs2282679 genetic variant of the CG gene was genotyped using DNA obtained from oral mucosa cells for all participants prior to bariatric surgery. Blood samples were taken for biochemical analysis before surgery and at 3, 6, and 12 months post-surgery. Medical records included history of dyslipidemia, hypertension, diabetes mellitus, and deficiency in 25-hydroxyvitamin D levels (< 30 ng/ml) at corresponding time points. Anthropometric measurements were also documented. All premenopausal females with obesity have been undergoing robotic sleeve gastrectomy (SG) (Table 1). This Robotic Sleeve Gastrectomy was performed using the DaVinci X™ (Intuitive Surgical LTD, Oxford, UK) platform with 5 trocars (four 8-mm robotic trocars and one 12-mm trocar for AirSeal® iFS (CONMED, NY USA). The gastrocolic ligament was cut across with robotic Vessel Sealer Extend™ (Intuitive Surgical LTD, Oxford, UK). Greater curvature was cut across with Signia™ Stapling System (Medtronic, Minneapolis, USA) and Endo GIA™ with Tri-Staple™ Technology (Medtronic, Minneapolis, USA). A 36 Fr bougie was used for calibration.

**Table 1:** Presurgical characteristics of the premenopausal females with obesity.

Parameters	Basal time
subjects with severe obesity	66
subjects with BMI > 50 kg/m^2^	10
Deficit of 25-hydroxyvitamin D ( <30 nmol/L)	14.1%
Age (years)	41.3 ± 2.2
BMI (kg/m^2^)	42.1 ± 2.1

*BMI* Body mass index.

Severe obesity: BMI > 40 kg/m^2^ and <50 kg/m^2^.

For the first month, the females with obesity received a 1000-calorie diet supplemented with a protein module to reach 1.4 g per Kg of ideal weight (Body Mass Index (BMI) 22 kg/m^2^) and the following distribution of macronutrient (20% protein, 30% fat and 70% carbohydrates). After one month of sleeve gastrectomy, subjects with obesity followed the same diet based on the intake of 1200-1400 calories, with the next distribution of macronutrients fats (35%, divided into 10% saturated, 20% monounsaturated and 5% polyunsaturated) and carbohydrates (65%), with a contribution of proteins of 1.2 g per Kg of ideal weight. Post-surgery, all patients received oral standard vitamin D supplement (1200 UI/day). If serum levels of 25-OH vitamin D were below 30 nmol/L at follow-up visits (+3, +6, +12 months) patients received an extra daily dose of 600 UI/day.

### Anthropometric measurements, blood pressure and comorbidities

In all cases, this data was collected in the morning before breakfast initially and then at 3, 6, and 12 months following the surgery; waist circumference was determined using a flexible standard tape (Omrom, LA, CA). Height (cm) was measured using a standard height measurement scale (Omrom, LA, CA, USA). Body weight were measured while the subjects were minimally unclothed and not wearing shoes, using digital scales (Omrom, LA, CA, USA). Body mass index (BMI) was obtained as weight in kilograms divided by height in squared meters. Ideal weight was calculated with an ideal BMI 22 kg/m^2^. Fat mass was measured by bioimpedance with an accuracy of 5 g [13] (EFG BIA 101 Anniversary, Akern, It).

Blood pressure was determined twice using a sphygmomanometer (Omrom, LA, CA, USA), after the subjects sat for 10 min.

Comorbidities of obesity were defined as high levels of triglycerides (triglycerides above or equal 150 mg/dl), hypertension (systolic and diastolic blood pressures above or equal than 140 and/or 90 mmHg, respectively), high levels of LDL-cholesterol (above or equal 100 mg/dl) or subjects who were taking medicament for any previous-mentioned entities. To determine diabetes mellitus, any of the next data were necessary (fasting blood glucose > 126 mg/dl or HBa1c > 6.5% or blood glucose after two-hour oral glycose overload test greater than 200 mg/dl) or females who were under treatment with any drugs for hyperglycemia.

### Biochemical assays and genotyping

In each visit, a 5 ml of venous blood after a 10 h overnight fast were aliquoted in ethylenediaminetetraacetic acid (EDTA)-coated tubes. Biochemical measurements, including glucose, insulin, C reactive protein (CRP), calcium, phosphorus, total cholesterol, HDL-cholesterol, and triglyceride levels were analysed using the COBAS INTEGRA 400 analyser (Roche Diagnostic, Basel, Switzerland). The homeostasis model assessment for insulin resistance (HOMA-IR) was evaluated using these values with the next equation; (glucose x insulin/22.5) [14]. LDL cholesterol was calculated using Friedewald formula (LDL cholesterol= total cholesterol-HDL cholesterol-triglycerides/5) [15, 16]. 25-hydroxy vitamin D was determined by Cobas 6000e-60 chemiluminescence immunoassay (Roche Diagnostic, Basel, Switzerland). Deficiency of 25-hydroxyvitamin D was considered < 30 ng/dl.

Genomic DNA was extracted from cells of the oral mucosa using QIAamp ®. DNA polymerase chain reaction (qPCR) was used to evaluate the polymorphism rs2282679 with the QuantStudio 12 K Flex Real-Time qPCR instrument (Thermofisher, Pittsburg, Pens, USA). A total volume of 15 μl with 2.5 μl TaqMan OpenArray Master Mix (Applied Biosystems, Foster City, LA, CA, USA) and 2.5 μl human DNA sample were loaded and amplified (Termocicler Life Technologies, LA, CA). Genotype calling and sample clustering for Open Array assays was performed in TaqMan Genotyper (Life Technologies, Carlsbad, CA, USA). Twenty percent of samples underwent repeat genotyping to ensure reproducibility. Moreover, a negative control and control samples representing all genotypes were included in each reaction. Hardy Weinberg equilibrium was evaluated with a statistical test (Chi-square). The variant of *CG* gene was in Hardy Weinberg equilibrium (p = 0.36).

### Statistical analysis

Sample size was determined to detect differences over 10 ng/dl of 25-hydroxyvitamin D levels with 90% power and 5% significance (n = 75). Bonferroni test was applied for multiple testing to reduce Type I error in association analysis. The *CG* rs2282679 genotype was analyzed using a dominant model (TT vs. TG + GG). Descriptive statistics of all variable values are presented as mean±standard deviation for continuous variables and as a percentage for categorical variables. Variables were analyzed with ANOVA test with Bonferroni posthoc test and Student t test (for normally distributed variable) or Kruskal-Wallis test (for non-normally-distributed variable). Odds ratio (OR) and 95% confidence interval (CI) was used to estimate the association of the rs2282679 SNV with 25-hydroxyvitamin D deficiency. Statistical analysis was performed using software SPSS for Windows, version 23.0 software package (SPSS Inc. Chicago, IL). P values below 0.05 were considered statistically significant.

## Results

A total of 76 premenopausal females with severe obesity received robotic sleeve gastrectomy. Before surgery, the parameters distribution is showed in Table 1. The allelic frequency was the following 0.76 T and 0.24 G alleles.

The genotypic frequency was the following; 50% (38 subjects) in TT genotype, 43.4% (33 subjects) in TG genotype and 6.6% (5 subjects) in GG genotype. We grouped the subjects in two groups; those carriers G allele (TG + GG, 50%) and non-carriers G allele (TT, 50%). Average age was similar in both genotype groups (TT: 42.1 ± 4.0 years *vs*. TG + GG 41.1 ± 3.2 years: ns), too.

Table 2 demonstrates alterations in anthropometric measurements and blood pressure levels with each follow-up after robotic sleeve gastrectomy. Anthropometric measurements↵and blood pressure at baseline were comparable in both genotypes. Upon reviewing the progression of these measures, a significant enhancement in systolic and diastolic blood pressure, body weight, and waist circumference was observed post-surgery at 3, 6, and 12 months. These enhancements were consistent across both genotypes (TT vs TG + GG). Additionally, improvement in blood pressure remained similar between the two groups over the course of the study.

**Table 2:** Changes in anthropometric variables Rs2282679 (Mean ± S.D).

Characteristics								
	TT (*n* = 38)				TG *or GG* (*n* = 38)			
	0 time	At 3 months	At 6 months	At 12 months	0 time	At 3 months	At 6 months	At 12 months
BMI	42.3 ± 3.0	36.1 ± 3.2^*^	31.1 ± 2.2^*^	30.9 ± 2.1^*^	46.1 ± 2.9	40.4 ± 2.1^*^	33.5 ± 2.1^*^	32.9 ± 2.2^*^
Weight (kg)	111.6 ± 9.2	95.2 ± 6.3^*^	87.1 ± 5.0^*^	84.1 ± 2.2^*^	110.8 ± 3.2	96.1 ± 3.2^*^	88.2 ± 3.0^*^	85.0 ± 2.1^*^
WC (cm)	114.1 ± 4.2	101.9 ± 2.1^*^	96.9 ± 3.9^*^	92.8 ± 3.1^*^	115.7 ± 3.0	102.1 ± 2.0^*^	97.1 ± 3.0^*^	91.3 ± 2.1^*^
SBP (mmHg)	141.1 ± 3.2	136.2 ± 2.8^*^	129.1 ± 3.2^*^	121.2 ± 3.1^*^	140.2 ± 4.1	135.9 ± 2.2^*^	128.1 ± 2.1^*^	121.1 ± 2.0^*^
DBP (mmHg)	88.2 ± 2.0	87.0 ± 3.1	83.1 ± 2.2^*^	83.0 ± 2.1^*^	91.0 ± 3.1	86.6 ± 2.0	83.2 ± 3.1*	82.1 ± 2.0^*^

*DBP* Diastolic blood pressure, *SBP* Systolic blood pressure, *WC* Waist circumference. (*) *p* < 0.05, in each genotype group with baseline data. There are no statistical differences in demographic, anthropometric and metabolic characteristics between the two-genotype groups.

Table 3 reports modifications in biochemical parameters after robotic sleeve gastrectomy. Basal values of these parameters are similar in both genotype groups except insulin and 25-hydroxyvitamin D levels. Basal insulin levels and HOMA-IR were greater in G allele carriers than non-carriers (Delta: 6.7 ± 1.2 mUI/L; *p* = 0.01) and (Delta: 1.3 ± 0.1 units; *p* = 0.02), respectively. On the contrary, 25 OH vitamin D levels were lower in G allele carriers than non-carriers (Delta: 8.1 ± 1.1 ng/dl; *p* = 0.03). The levels of insulin and HOMA-IR remained greater in G allele carriers than non-carriers throughout all the visits. The levels of 25-hydroxyvitamin D remained lower in G allele carriers than non-G allele during all the study. In the other hand, the level of 25-hydroxyvitamin D at 12 months in non-G allele carriers were above 30 ng/dl (36.0 ± 3.1 ng/dl) and the level in G allele carriers were below 30 ng/dl (24.9 ± 4.9 ng/dl). As expected, the important amount of weight loss produced a significant decrease of fasting glucose, insulin, HOMA-IR, total cholesterol, LDL-cholesterol and triglyceride levels in both genotype groups in each visit.

**Table 3:** Biochemical Parameters Rs2282679 (Mean ± S.D).

Characteristics								
	TT (*n* = 38)				TG *or GG* (*n* = 38)			
	0 time	At 3 months	At 6 months	At 12 months	0 time	At 3 months	At 6 months	At 12 months
Glucose (mg/dl)	115.8 ± 4.2	92.1 ± 3.2^*^	91.3 ± 2.1^*^	88.0 ± 3.1^*^	119.9 ± 4.1	94.9 ± 4.0^*^	91.1 ± 2.1^*^	89.1 ± 3.0^*^
Total ch. (mg/dl)	199.3 ± 8.2	163.2 ± 7.1^*^	159.2 ± 6.1^*^	158.2 ± 4.2^*^	200.1 ± 7.1	164.1 ± 6.3^*^	160.7 ± 5.1^*^	157.7 ± 3.0^*^
LDL-ch. (mg/dl)	134.3 ± 7.1	105.9 ± 6.1^*^	101.2 ± 3.2^*^	97.2 ± 3.1^*^	135.9 ± 6.0	104.1 ± 3.1^*^	102.0 ± 3.0^*^	96.8 ± 3.2^*^
HDL-ch. (mg/dl)	46.2 ± 3.0	45.9 ± 2.4	44.7 ± 4.0	45.0 ± 3.0	46.2 ± 3.2	45.1 ± 2.2	44.7 ± 3.1	45.1 ± 630
TG (mg/dl)	144.4 ± 9.1	125.1 ± 8.2^*^	111.7 ± 7.6^*^	105.4 ± 8.1^*^	151.1 ± 10.9	125.1 ± 8.1^*^	110.2 ± 8.1*	104.7 ± 5.2^*^
Insulin (mUI/L)	18.2 ± 2.1	12.0 ± 2.0^*^	7.8 ± 2.3^*^	7.9 ± 1.8^*^	24.9 ± 2.0^$^	16.1 ± 1.9^*,$^	12.2 ± 3.0^*,$^	11.9 ± 2.1^*,$^
HOMA-IR	4.6 ± 1.1	2.4 ± 0.5*	2.0 ± 0.4^*^	1.6 ± 0.3^*^	5.9 ± 0.8^$^	3.9 ± 0.9^*,$^	3.1 ± 0.6^*,$^	2.5 ± 0.4^*,$^
25-hydroxyvitamin D (ng/dl)	25.2 ± 2.1	26.1 ± 2.2	27.9 ± 2.0	36.0 ± 3.1^*^	17.9 ± 2.2^$^	18.1 ± 3.1^$^	20.2 ± 4.1^$^	24.9 + 4.9^$^

*LDL* Low density lipoprotein, *HDL* High density lipoprotein, *Chol* Cholesterol, *TG* Triglycerides, *HOMA-IR* homeostasis model assessment. Season blood collection (winter, spring, summer and autumn were similar in both genotype groups). *p < 0.05, in each group with baseline values. ^$^*p* < 0.05 statistical differences between genotypes.

Table 4 demonstrates the enhancement in obesity-related complications over the course of the research (percentage of elevated triglycerides, hypertension, high-LDL cholesterol levels, and type 2 diabetes mellitus) as well as the percentage of deficiency in 25-hydroxyvitamin D. The initial percentages of type 2 diabetes mellitus and deficiency in 25-hydroxyvitamin D were higher in G allele carriers than non-carriers. These variances persisted throughout the study.

**Table 4:** Percentage Of Obesity Comorbidities And Deficit Of 25 Oh Vitamin D.

Parameters	Baseline	3 months	6 months	12 months
**High levels LDL Cholesterol**				
TT	39.4%	26.3%	21.1%*	18.4%*
TG + GG	42.1%	26.3%	18.4%*	18.4%*
**High Levels TG**				
TT	18.4%	15.8%	7.9%*	7.9%*
TG + GG	15.8%	15.8%	5.3%*	5.3%*
**Blood Hypertension**				
TT	23.6%	15.8%*	15.8%*	15.8%*
TG + GG	26.3%	18.4%*	15.8%*	10.5%*
**Diabetes mellitus**				
TT	13.1%	10.5%	10.5%	7.9%*
TG + GG	34.2%^$^	31.8%^$^	28.9%^$^	26.3%^$^
**Deficit 25-hydroxyvitamin D**				
TT	21.1%	13.2%	10.6%	7.9%*
TG + GG	42.1%^$^	31.6%^$^	26.3%^$^	23.6%^$^

HyperTG Hypertriglyceridemia (triglycerides >150 mg/dl), hypertension (systolic and diastolic blood pressures greater than 130 and 85 mmHg respectively), High LDL cholesterol (>100 mg/dl). Deficit 25 OH Vitamin D ( < 30 ng/dl). Season blood collection (winter, spring, summer and autumn were similar in both genotype groups).

*p < 0.05, in each group with basal values. ^$^*p* < 0.05 statistical differences between genotypes.

Percentage of diabetes mellitus decreased in a significant way in non-G allele carriers in the last visit (from 13.1% to 7.9%; *p* = 0.04), the improvement in diabetes percentage in G allele carriers did not reach statistical significance. Deficit of 25-hydroxyvitamin D improved in a significant way in non-G allele carriers in the last visit (21.1% vs 5.2%; *p* = 0.03), the decrease of 25-hydroxyvitamin D percentage in G allele carriers were not significant. According to the results, at baseline the percentages of individuals who had deficit of 25-hydroxyvitamin D (OR = 2.75, 95% CI = 1.01–791; *p* = 0.01) and percentage of diabetes mellitus (OR = 3.41, 95% CI = 1.08–10.8; *p* = 0.02) were greater in G allele carriers than non-G allele subjects. At the last visit (12 months), percentage of deficit of 25-hydroxyvitamin D (OR = 5.59, 95% CI = 1.12–27.97; *p* = 0.03) and percentage of diabetes mellitus (OR = 4.17, 95% CI = 1.95-16.61; *p* = 0.03) remained greater in G allele carriers, too.

## Discussion

Premenopausal women with obesity and the G allele of the rs2282679 SNV demonstrated reduced 25-hydroxyvitamin D levels and higher insulin levels, as well as increased insulin resistance. Furthermore, the presence of the G allele hindered improvement in 25-hydroxyvitamin D levels and insulin resistance following significant weight loss due to robotic sleeve gastrectomy.

The literature contains limited and conflicting studies that have examined the impact of single nucleotide variations on 25-hydroxyvitamin D levels. Additionally, there is a lack of research evaluating the role of rsThe research on the impact of SNV on 25-hydroxyvitamin D levels is limited and conflicting. There are no studies that have specifically assessed the role of rs2282679 after bariatric surgery. Some studies in the literature report a lack of association [17, 18], while others indicate a relationship between certain SNVs and 25-hydroxyvitamin D [8, 19]. Moreover, some investigations have reported, specifically in rs2282679, a relationship with vitamin D deficiency in different populations; Caucasians [20, 21] and Asiatic [22]. This SNV is located in *GC* gene that encode vitamin D-binding protein (DBP). This protein joints to VD sterol metabolites for transporting them through the blood circulation towards targets organs, and previous studies have reported that levels of vitamin D metabolites were associated with serum DBP levels [23, 24]. This considerable reduction in DBP levels in circulation could hypothetically account for the link between this SNV and the reported 25-hydroxyvitamin D levels in our intervention study.

Additionally, we discovered a connection between rs2282679 and HOMA-We also discovered a connection of rs2282679 with HOMA-IR and fasting insulin levels. In fact, lower circulating levels of 25 hydroxyvitamin D in females during premenopause [25] have been linked to insulin resistance, and this association has been attributed to various mechanisms. It is plausible that the notable decrease in circulating DBP levels could account for the link between this SNV and reported 25-hydroxyvitamin D levels in our interventional study. Firstly, 25-hydroxyvitamin D induces insulin secretion in pancreatic Beta-cell [26]. Secondly, 25-hydroxyvitamin D modulates insulin sensitivity in fat and muscle tissues by regulating extracellular calcium levels, this cation is necessary for insulin–mediated intracellular processes [27]. Finally, low levels of 25-hydroxyvitamin D might induce an inflammatory status, which is associated with insulin resistance, too [28]. Therefore, these pathophysiological pathways may account for our metabolic findings due to lower levels of vitamin D in G allele carriers. Although we did not find any link between rs2282679 and adiposity data in our presurgical analysis, Wehr et al. [29] identified a correlation with waist circumference in premenopausal females with polycystic ovary syndrome. The underlying mechanism behind this association remains unknown but warrants further investigation.

On the other hand, numerous factors could influence bariatric surgery and lead to highly diverse results in terms of responses to associated health conditions [30, 31]. In our research, while we found no variances in initial weight and weight changes based on genotypes, insulin levels and HOMA-IR were higher in G allele carriers compared to non-G allele carriers after 12 months of robotic sleeve gastrectomy. Previous studies have reported that insulin resistance improvements after bariatric surgery are related with weight loss [32] and a second hypothesis, it is that sleeve gastrectomy may improve insulin action via gut hormone modification [32], too. However, the different observed levels of 25-hydroxyvitamin D may also influence the reported outcomes of insulin resistance, as above-mentioned [26–28]. In addition, the relationships between 25-hydroxyvitamin D levels and bariatric surgery are complex. Recently, Bandstein et al. [33] have reported that presurgical 25-hydroxyvitamin D levels modulate the magnitude of genotype effects of rs9939609 on Roux-en-Y Gastric Bypass Surgery induced weight loss. The excessive weight loss of vitamin D deficient subjects carrying AA exceeded that of vitamin D deficient subjects carrying TT genotype. This suggests that vitamin D may possess biological effect that can regulates the impact by which *FTO* gene or other genes impact body weight regulation [34].

Our study is limited in several ways. Firstly, the findings may not be applicable to the general population as the research was conducted in Caucasian premenopausal females with obesity. Secondly, we did not measure circulating levels of DBP or consider potential interactions it may have with genetic variants. Thirdly, there are other unknown non-genetic factors such as exercise, hormone status, socioeconomic status, and epigenetic factors that could influence our results. Additionally, a lack of dietary assessment among subjects with obesity might introduce bias into our findings. Finally, not having an untreated group without bariatric surgery could also pose a bias in our study design. On the positive side though - this real-life study incorporates active vitamin D supplementation and nutritional monitoring for patients which has been shown beneficial especially for carriers of the G allele.

In summary, the hereditary variation of rs 2282679 was linked to levels of 25-hydroxyvitamin D and insulin resistance in Caucasian premenopausal women with obesity undergoing robotic sleeve gastrectomy. Moreover, the presence of G allele hindered the improvement in 25-hydroxyvitamin D levels and insulin resistance after weight loss over a period of 12 months, despite standard vitamin D supplementation. Our findings suggest that it may be necessary to genotype these patients before bariatric surgery in order to enhance their vitamin D supplementation. However, further studies are required to confirm the impact of vitamin D on changes in insulin resistance.

## Data Availability

All data generated or analyzed during this study are included in this article. Further enquiries can be directed to the corresponding author.

## References

[1] Hilger J, Friedel A, Herr R, Rausch T, Roos F, Pierroz D (2014). A systematic review of vitamin D status in populations worldwide. Br J Nutr.

[2] Looker AC (2005). Body fat and vitamin D status in black *versus* white women. J Clin Endocrinol Metab.

[3] de Luis DA, Izaola O, Primo D (2021). The lactase rs4988235 is associated with obesity related variables and diabetes mellitus in menopausal obese females. Eur Rev Med Pharm Sci.

[4] de Luis DA, Izaola O, Primo D, de la Fuente B, Aller R (2017). Polymorphism rs3123554 in the cannabinoid receptor gene type 2 (CNR2) reveals effects on body weight and insulin resistance in obese subjects. Endocrinol Diabetes Nutr.

[5] Snellman G, Melhus H, Gedeborg R, Olofsson S, Wolk A, Micahelsoon K (2009). Seasonal genetic influence on serum 25- hydroxyvitamin D levels: a twin study. PLoS One.

[6] Wang TJ, Zhang F, Richards JB, Kestenbaum B, Van Meurs JB, Berry D (2010). Common genetic determinants of vitamin D insufficiency: a genome-wide association study. Lancet.

[7] Aydin Y, Hassa H, Burkankulu D, Arslantas D, Sayiner D, Ozerdogan N (2015). What is the Risk of Metabolic Syndrome in Adolescents with Normal BMI who have Polycystic Ovary Syndrome?. J Pediatr Adolesc Gynecol.

[8] Sinotte M, Diorio C, Berube S, Pollak M, Brisson J (2009). Genetic polymorphisms of the vitamin D binding protein and plasma concentrations of 25-hydroxyvitamin D in premenopausal women. Am J Clin Nutr.

[9] Gozdzik A, Zhu J, Wong BYL, Fu L, Cole DEC, Parra EJ (2011). Association of vitamin D binding protein (VDBP) polymorphisms and serum 25(OH)D concentrations in a sample of young Canadian adults of different ancestry. J Steroid Biochem Mol Biol.

[10] Sarwer DB, Steffen KJ (2015). Quality of life, body image and sexual functioning in bariatric surgery patients. Eur Eat Disord Rev.

[11] Cui B, Zhu L, Zhu S (2022). Effects of Roux-en-Y Gastric Bypass Versus Sleeve Gastrectomy on Body Composition for Patients with a BMI > 35 kg/m2 at 1 Year After Surgery. Obes Surg.

[12] Pacheco D, Izaola O, Primo D, de Luis D (2022). Role of the variant rs3774261 of adiponectin gene on adiponectin levels and ratio adiponectin/leptin after biliopancreatic diversion in morbid obese subjects. Eur Rev Med Pharm Sci.

[13] Toribio MJ, Priego-Capote F, Pérez-Gómez B, Fernández de Larrea-Baz N, Ruiz-Moreno E, Castelló A (2021). Factors Associated with Serum Vitamin D Metabolites and Vitamin D Metabolite Ratios in Premenopausal Women. Nutrients.

[14] Lukaski H, Johson PE (1985). Assessment of fat-free mass using bioelectrical impedance measurements of the human body. Am J Clin Nutr.

[15] Mathews DR, Hosker JP, Rudenski AS, Naylor BA, Treacher DF (1985). Homeostasis model assessment: insulin resistance and beta cell function from fasting plasma glucose and insulin concentrations in man. Diabetologia.

[16] Friedewald WT, Levy RJ, Fredrickson DS (1972). Estimation of the concentration of low-density lipoprotein cholesterol in plasma without use of the preparative ultracentrifuge. Clin Chem.

[17] Almesri N, Das NS, Ali ME, Gumaa K, Giha HA (2016). Independent association of polymorphisms in vitamin D binding protein (GC) and vitamin D receptor (VDR) genes with obesity and plasma 25OHD3 levels demonstrate sex dimorphism. Appl Physiol, Nutr, Metab.

[18] Yao S, Hong CC, Bandera EV, Zhu Q, Liu S, Cheng TD (2017). Demographic, lifestyle, and genetic determinants of circulating concentrations of 25-hydroxyvitamin D and vitamin D-binding protein in African American and European American women. Am J Clin Nutr.

[19] Abbas S, Nieters A, Linseisen J, Slanger T, Kropp S, Mutschelknauss EJ (2008). Vitamin D receptor gene polymorphisms and haplotypes and postmenopausal breast cancer risk. Breast Cancer Res.

[20] Nissen J, Vogel U, Ravn-Haren G, Andersen EW, Madsen KH, Nexo B (2015). Common variants in CYP2R1 and GC genes are both determinants of serum 25-hydroxyvitamin D concentrations after UVB irradiation and after consumption of vitamin D(3)-fortified bread and milk during winter in Denmark. Am J Clin Nutr.

[21] Batai K, Murphy AB, Shah E, Ruden M, Newsome J, Agate S (2014). Common vitamin D pathway gene variants reveal contrasting effects on serum vitamin D levels in African Americans and European Americans. Hum Genet.

[22] Li LH, Yin XY, Wu XH, Zhang L, Pan SY, Zheng Z (2014). *S*erum 25(OH)D and vitamin D status in relation to VDR,GC and CYP2R1 variants in Chinese. Endocr J.

[23] Hansen JG, Tang W, Hootman KC, Brannon PM, Houston DK, Kritchevsky SB (2015). Genetic and environmental factors are associated with serum 25-hydroxyvitamin D concentrations in older African Americans. J Nutr.

[24] Tint GS, Irons M, Elias ER, Batta AK, Frieden R, Chen TS (1994). Defective cholesterol biosynthesis associated with the Smith-Lemli-Opitz syndrome. N. Engl J Med.

[25] Giovinazzo S, Alibrandi A, Campennı A, Trimarchi F, Ruggeri RM (2017). Correlation of cardio-metabolic parameters with vitamin D status in healthy premenopausal women. J Endocrinol Invest.

[26] Bland R, Markovic D, Hills CE, Hughes SV, Chan SL, Squires PE (2004). Expression of 25-hydroxyvitamin D3-1alpha-hydroxylase in pancreatic islets. J Steroid Biochem Mol Biol.

[27] Pittas AG, Lau J, Hu FB, Dawson-Hughes B (2007). The role of vitamin D and calcium in type 2 diabetes. A systematic review and meta-analysis. J Clin Endocrinol Metab.

[28] Ginsberg HN, Zhang YL, Hernandez-Ono A (2005). Regulation of plasma triglycerides in insulin resistance and diabetes. Arch Med Res.

[29] Wehr E, Trummer O, Giuliani A, Gruber HJ, Pieber TR, Obermayer-Pietsch B (2011). Vitamin D-associated polymorphisms are related to insulin resistance and vitamin D deficiency in polycystic ovary syndrome. Eur J Endocrinol.

[30] Dzamko N, van Denderen BJ, Hevener AL, Jørgensen SB, Honeyman J, Galic S (2010). AMPK beta1 deletion reduces appetite, preventing obesity and hepatic insulin resistance. J Biol Chem.

[31] Saiki A, Olsson M, Jernås M, Gummesson A, McTernan PG, Andersson J (2009). Tenomodulin is highly expressed in adipose tissue, increased in obesity, and down-regulated during diet-induced weight loss. J Clin Endocrinol Metab.

[32] Toledo FG, Menshikova EV, Ritov VB, Azuma K, Radikova Z, DeLany J (2007). Effects of physical activity and weight loss on skeletal muscle mitochondria and relationship with glucose control in type 2 diabetes. Diabetes.

[33] Bandstein M, Schultes B, Ernst B, Thurnheer M, Schiöth HB, Benedict C (2015). The Role of FTO and Vitamin D for the Weight Loss Effect of Roux-en-Y Gastric Bypass Surgery in Obese Patients. Obes Surg.

[34] Dusso AS, Brown AJ, Slatopolsky E (2005). Vitamin D. Am J Physiol Ren Physiol.

